# Supplementary material for: Sinus Tarsi Morphometry Is Correlated with Flatfoot Severity on Weight-Bearing CT
Source: Diagnostics (Basel). 2026 Jan 4;16(1):162. doi: 10.3390/diagnostics16010162 (PMC12785349; doi:10.3390/diagnostics16010162)
Supplement: Supplementary file 1 [file diagnostics-16-00162-s001.zip › diagnostics-3987869-supplementary.pdf]

Supplementary Table S1. Intraclass correlation coefficient (ICC) with 95% confidence interval (95% CI) for assessing inter- and intra-observer agreement in the measurement of seven parameters on weight-bearing X-ray and CT images.

	Inter-observer agreement [ICC (95% CI)]	Intra-observer agreement [ICC (95% CI)]		
		Observer 1	Observer 2	Observer 3
Meary angle (°)	0.805 (0.678–0.881)	0.903 (0.618–0.961)	0.944 (0.867–0.972)	0.882 (0.629–0.949)
Pitch angle (°)	0.875 (0.822–0.916)	0.947 (0.914–0.967)	0.970 (0.953–0.981)	0.966 (0.946–0.979)
STL (mm)	0.821 (0.503–0.919)	0.935 (0.562–0.978)	0.936 (0.900–0.960)	0.939 (0.895–0.963)
STW (mm)	0.845 (0.774–0.897)	0.947 (0.912–0.968)	0.926 (0.884–0.953)	0.929 (0.875–0.958)
ST-H angle (°)	0.793 (0.710–0.858)	0.923 (0.797–0.964)	0.912 (0.862–0.945)	0.906 (0.853–0.941)
ST angle (°)	0.886 (0.710–0.945)	0.958 (0.880–0.980)	0.962 (0.916–0.980)	0.960 (0.912–0.979)
Tibia width (mm)	0.847 (0.607–0.927)	0.927 (0.816–0.964)	0.917 (0.864–0.949)	0.927 (0.885–0.954)

Supplementary Table S2. Regression coefficients of variables in each stepwise model (Meary angle as dependent variable).

**Meary angle as dependent variable.**						
		Unstandardized		Standardized		
		B	SER	β	t	*p* value
Model 1	Intercept	11.819	1.260		9.382	**<0.001**
	ST angle (°)	-0.259	0.069	-0.416	-3.767	**<0.001**
Model 2	Intercept	-0.389	4.748		-0.082	0.935
	ST angle (°)	-0.212	0.068	-0.339	-3.096	**0.003**
	STL/Tibia width	13.340	5.018	0.291	2.659	**0.010**
Model 3	Intercept	6.478	4.916		1.318	0.192
	Gender*	-5.098	1.567	-0.344	-3.254	**0.002**
	ST angle (°)	-0.180	0.065	-0.289	-2.789	**0.007**
	STL/Tibia width	8.825	4.894	0.193	1.803	0.076

ST: sinus tarsi, STL: sinus tarsi length. *: female=0, male=1. Bolded *p* values indicate statistical significance at $p<0.05$.

Supplementary Table S3. Excluded variables in linear regression analysis (Meary angle as dependent variable).

	Predictive variable	Input β	t	*p* value	Partial Correlation	Tolerance
Model 1	Gender*	-0.398	-3.862	**<0.001**	-0.427	0.951
	Height (m)	-0.325	-2.931	**0.005**	-0.337	0.888
	STL (mm)	0.162	1.447	0.153	0.174	0.954
	STL/Tibia width	0.291	2.659	**0.010**	0.309	0.931
Model 2	Gender*	-0.344	-3.254	**0.002**	-0.372	0.875
	Height (m)	-0.278	-2.530	**0.014**	-0.297	0.855
	STL (mm)	-0.077	-0.504	0.616	-0.062	0.487
Model 3	Height (m)	-0.075	-0.506	0.614	-0.063	0.453
	STL (mm)	0.085	0.562	0.576	0.070	0.434

STL: sinus tarsi length. *: female=0, male=1. Bolded *p* values indicate statistical significance at $p<0.05$.

Supplementary Table S4. Regression coefficients of variables in each stepwise model (Pitch angle as dependent variable).

**Pitch angle as dependent variable.**						
		Unstandardized		Standardized		
		B	SER	β	t	*p* value
Model 1	Intercept	15.337	1.195		12.830	**<0.001**
	ST angle (°)	0.248	0.065	0.418	3.791	**<0.001**
Model 2	Intercept	28.270	4.446		6.358	**<0.001**
	ST angle (°)	0.197	0.064	0.332	3.079	**0.003**
	STL/Tibia width	-14.134	4.699	-0.325	-3.008	**0.004**

ST: sinus tarsi, STL: sinus tarsi length, β: standardized regression coefficient. Bolded *p* values indicate statistical significance at $p<0.05$.

Supplementary Table S5. Excluded variables in linear regression analysis (Pitch angle as dependent variable).

Model	Predictive variable	Input β	t	*p* value	Partial Correlation	Tolerance
Model 1	Gender*	0.258	2.354	**0.021**	0.276	0.951
	Height (m)	0.223	1.946	0.056	0.231	0.888
	STL (mm)	-.0285	-2.633	**0.011**	-0.306	0.954
	STL/Tibia width	-0.325	-3.008	**0.004**	-0.345	0.931
Model 2	Gender*	0.181	1.645	0.105	0.198	0.875
	Height (m)	0.165	1.477	0.144	0.179	0.855
	STL (mm)	-0.118	-0.790	0.433	-0.097	0.487

STL: sinus tarsi length. *: female=0, male=1. Bolded *p* values indicate statistical significance at $p<0.05$.

Supplementary Table S6. Regression ANOVA in each stepwise model (Meary angle as dependent variable).

Meary angle as dependent variable.						
		Sum of square	df	Mean square	F	*p* value
	Regression	585.985	1	585.985	14.192	**<0.001**
Model 1	Residual	2807.786	68	41.291		
	Total	3393.771	69			
	Regression	853.928	2	426.964	11.263	**<0.001**
Model 2	Residual	2539.844	67	37.908		
	Total	3393.771	69			
	Regression	1204.994	3	401.665	12.112	**<0.001**
Model 3	Residual	2188.778	66	33.163		
	Total	3393.771	69			

Bolded *p* values indicate that the overall regression model is statistically significant at $p<0.05$.

Supplementary Table S7. Regression ANOVA in each stepwise model (Pitch angle as dependent variable).

Pitch angle as dependent variable.						
		Sum of square	df	Mean square	F	*p* value
	Regression	534.335	1	534.335	14.371	**<0.001**
Model 1	Residual	2528.308	68	37.181		
	Total	3062.643	69			
	Regression	835.110	2	417.555	12.559	**<0.001**
Model 2	Residual	2227.533	67	33.247		
	Total	3062.643	69			

Bolded *p* values indicate that the overall regression model is statistically significant at $p<0.05$.

Supplementary Table S8. Model summary.

Dependent variable	Model	R	R^2	Adjusted R^2	SER
Meary angle (°)	1	0.416	0.173	0.160	6.426
	2	0.502	0.252	0.229	6.157
	3	0.596	0.355	0.326	5.759
Pitch angle (°)	1	0.418	0.174	0.162	6.098
	2	0.522	0.273	0.251	5.766

SER: Standard error of the regression.
